# Draft Genome Sequences of Bacillus velezensis Strains AF_3B and OS2, Bacillus amyloliquefaciens Strain BS9, Bacillus halotolerans Strain A1, and *Bacillus* sp. Strain BS3, Producing Biosurfactants with Antimicrobial Potential

**DOI:** 10.1128/mra.00482-22

**Published:** 2022-09-21

**Authors:** Syeda Amna Farooq, Anne de Jong, Shazia Khaliq, Oscar P. Kuipers

**Affiliations:** a Industrial Biotechnology Division, National Institute for Biotechnology and Genetic Engineering (NIBGE), Constituent College, Pakistan Institute of Engineering and Applied Sciences (PIEAS), Faisalabad, Pakistan; b Department of Molecular Genetics, University of Groningen, Groningen, The Netherlands; University of Maryland School of Medicine

## Abstract

Five environmental *Bacillus* strains were sequenced, of which three were isolated from the rhizosphere of agricultural soil and one each from Attock Oil Refinery and Khewra Salt Mine in Pakistan. The strains can be used for plant growth promotion and biosurfactant activity brought about by secondary metabolites.

## ANNOUNCEMENT

The demand for biosurfactants is rising, and members of the genus *Bacillus* are the pivotal in their production (1). Biosurfactants can lower the critical micelle concentration and surface tension of water (2–4). They can replace synthetic surfactants due to their strong antimicrobial potential against a range of pathogens (5).

Bacilli (6) are well-studied producers of secondary metabolites, including lipopeptides, bacteriocins, terpenes, polyketides, and lanthipeptides, used as biocontrol for plant growth promotion. Almost, 4 to 5% of the genome of *Bacillus* species is responsible for the production of these antagonistic metabolites (6, 7).

Five potential *Bacillus* strains were isolated from various regions of Pakistan. Two strains (AF_3B and A1) were isolated from the soil rhizosphere around the roots of wheat plants, and strain BS9 was isolated from the rhizosphere around the roots of the cotton plants. The other two strains (OS2 and BS3) were isolated from oily sludge at Attock petroleum oil refinery and salty water collected from Khewra Salt Mine in Pakistan, respectively. The soil and oil samples collected were dissolved in sterile distilled water and incubated at 37°C for 24 h until the optical density at 600 nm (OD_600_) was observed to be above 0.5. The samples were serially diluted; 1 mL of a 10^9^ dilution was spread onto LB agar plates and incubated at 37°C for 48 h. Bacterial colonies on agar plates were selected on the basis of apparent *Bacillus*-like colony morphology, circular, rough, opaque, or slightly yellowish with lifted edges. Each selected colony was monitored using the DeltaVision system (Applied Precision, Washington) with an IX71 phase contrast microscope (Olympus, PA, USA) for further confirmation of spore-forming rod-shaped cell morphology.

For genome sequencing, a single colony from the LB plate was cultured in LB broth and incubated at 37°C and 20 rpm for 16 h. Cells were harvested by centrifugation at 10,000 rpm for 20 min at 4°C (Beckman Coulter Avanti centrifuge), and DNA extraction was performed using the manufacturer’s protocol with the GenElute bacterial genomic DNA isolation kit (Sigma-Aldrich, Germany). The 16S rRNA genes of the five selected isolates were amplified using 5′-AGAGTTTGATCCTGGCTCAG-3′ and 5′-ACGGTACCTTGTTACGACTT-3′ as the forward and reverse primers, respectively (8), and sequenced, and a BLASTN analysis was performed to confirm that all five isolates belonged to the genus *Bacillus*. The sequences have been submitted to GenBank and accession numbers are given in Table 1.

**TABLE 1:** Genomic properties and accession numbers for five *Bacillus* strains

Bacterial strain	Genome size (bp)	G+C content (%)	No. of coding sequences	No. of reads	*N*_50_ (bp)	No. of contigs	Coverage (×)	No. of RNAs	GenBank accession no. for:		SRA accession no.
									Whole genome	16S rRNA gene	
Bacillus velezensis AF_3B	3,949,216	46.3	4,041	6,159,113	581,547	28	150	65	JAJIBB000000000	OL771696.1	SRX13297035
Bacillus velezensis OS2	3,968,045	46.3	4,054	6,183,522	418,400	32	154	65	JAJIBC000000000	OL771697.1	SRX13452167
Bacillus amyloliquefaciens BS9	3,949,726	46.3	4,034	6,158,224	428,550	30	152	65	JAJOLX000000000	OL757572.1	SRX13404315
Bacillus halotolerans A1	4,058,484	43.8	4,205	6,201,404	525,368	29	156	71	JAJEJN000000000	OL757644.1	SRX13187240
*Bacillus* sp. BS3	4,351,328	45.9	4,652	6,159,214	399,602	27	150	69	JAJNDM000000000	OL757643.1	SRX13347513

The genomes of the five screened strains were sequenced using an Illumina HiSeq sequencing system at BGI Tech Solutions (Hong Kong). For each sample, a minimum of 5 million high-quality paired-end raw reads (150 bp) were acquired (Table 1). The quality of the reads was accessed using FastQC version 0.11.9 (9), and contamination by low-quality reads was removed using Trimmomatic version 0.38 (10). The high-quality reads were assembled using Unicycler version 0.4.8 (11) embedded in SPAdes version 3.12.0 (12). All software was used with default parameters. The genome sequences were annotated by NCBI using the Prokaryotic Genome Annotation Pipeline (PGAP) (13) and identified by phylogenetic analysis as *Bacillus* spp. (13).

### Data availability.

The draft genome sequences of the five selected strains have been submitted to GenBank under the accession numbers listed in Table 1.

## References

[1] Théatre A, Cano-Prieto C, Bartolini M, Laurin Y, Deleu M, Niehren J, Fida T, Gerbinet S, Alanjary M, Medema MH, Leonard A, Lins L, Arabolaza A, Gramajo H, Gross H, Jacques P. 2021. The surfactin-like lipopeptides from Bacillus spp.: natural biodiversity and synthetic biology for a broader application range. Front Bioeng Biotechnol 9:623701. doi:10.3389/fbioe.2021.623701. 33738277 PMC7960918

[2] Hirata Y, Ryu M, Oda Y, Igarashi K, Nagatsuka A, Furuta T, Sugiura M. 2009. Novel characteristics of sophorolipids, yeast glycolipid biosurfactants, as biodegradable low-foaming surfactants. J Biosci Bioeng 108:142–146. doi:10.1016/j.jbiosc.2009.03.012. 19619862

[3] Arima K, Kakinuma A, Tamura G. 1968. Surfactin, a crystalline peptidelipid surfactant produced by Bacillus subtilis: isolation, characterization and its inhibition of fibrin clot formation. Biochem Biophys Res Commun 31:488–494. doi:10.1016/0006-291X(68)90503-2. 4968234

[4] Willenbacher J, Yeremchuk W, Mohr T, Syldatk C, Hausmann R. 2015. Enhancement of surfactin yield by improving the medium composition and fermentation process. AMB Express 5:145. doi:10.1186/s13568-015-0145-0. 26297438 PMC4546119

[5] Zhao H, Shao D, Jiang C, Shi J, Li Q, Huang Q, Rajoka MSR, Yang H, Jin M. 2017. Biological activity of lipopeptides from Bacillus. Appl Microbiol Biotechnol 101:5951–5960. doi:10.1007/s00253-017-8396-0. 28685194

[6] Stein T. 2005. Bacillus subtilis antibiotics: structures, syntheses and specific functions. Mol Microbiol 56:845–857. doi:10.1111/j.1365-2958.2005.04587.x. 15853875

[7] Fira D, Dimkic I, Beric T, Lozo J, Stankovic S. 2018. Biological control of plant pathogens by Bacillus species. J Biotechnol 285:44–55. doi:10.1016/j.jbiotec.2018.07.044. 30172784

[8] Srinivasan R, Karaoz U, Volegova M, MacKichan J, Kato-Maeda M, Miller S, Nadarajan R, Brodie EL, Lynch SV. 2015. Use of 16S rRNA gene for identification of a broad range of clinically relevant bacterial pathogens. PLoS One 10:e0117617. doi:10.1371/journal.pone.0117617. 25658760 PMC4319838

[9] Andrews S. 2010. FastQC: a quality control tool for high throughput sequence data. Babraham Bioinformatics, Babraham Institute, Cambridge, UK.

[10] Bolger AM, Lohse M, Usadel B. 2014. Trimmomatic: a flexible trimmer for Illumina sequence data. Bioinformatics 30:2114–2120. doi:10.1093/bioinformatics/btu170. 24695404 PMC4103590

[11] Wick RR, Judd LM, Gorrie CL, Holt KE. 2017. Unicycler: resolving bacterial genome assemblies from short and long sequencing reads. PLoS Comput Biol 13:e1005595. doi:10.1371/journal.pcbi.1005595. 28594827 PMC5481147

[12] Bankevich A, Nurk S, Antipov D, Gurevich AA, Dvorkin M, Kulikov AS, Lesin VM, Nikolenko SI, Pham S, Prjibelski AD, Pyshkin AV, Sirotkin AV, Vyahhi N, Tesler G, Alekseyev MA, Pevzner PA. 2012. SPAdes: a new genome assembly algorithm and its applications to single-cell sequencing. J Comput Biol 19:455–477. doi:10.1089/cmb.2012.0021. 22506599 PMC3342519

[13] Tatusova T, DiCuccio M, Badretdin A, Chetvernin V, Nawrocki EP, Zaslavsky L, Lomsadze A, Pruitt KD, Borodovsky M, Ostell J. 2016. NCBI Prokaryotic Genome Annotation Pipeline. Nucleic Acids Res 44:6614–6624. doi:10.1093/nar/gkw569. 27342282 PMC5001611

